# Trying again (and again): Weight cycling and depressive symptoms in U.S. adults

**DOI:** 10.1371/journal.pone.0239004

**Published:** 2020-09-11

**Authors:** Diane M. Quinn, Rebecca M. Puhl, Mora A. Reinka

**Affiliations:** 1 Department of Psychological Sciences, University of Connecticut, Storrs, Connecticut, United States of America; 2 University of Connecticut Rudd Center for Food Policy & Obesity, Department of Human Development and Family Sciences, University of Connecticut, Storrs, Connecticut, United States of America; Baylor University, UNITED STATES

## Abstract

As the prevalence of overweight and obesity have risen over the past few decades, so have weight control attempts. Research has shown, however, that intentional weight loss results are often short-lived, with people regaining the weight over time. This can lead to weight cycling–losing and gaining weight repeatedly. Previous research, mostly done over two decades ago, concluded there was no relationship between weight cycling and psychological health. The goal of the current paper was to re-examine the relationship between weight cycling and depressive symptoms in a national sample of American adults (*N* = 2702; 50.7% female; mean age = 44.8 years). If, as hypothesized, there is a relationship between more frequent weight cycling and depressive symptoms, then internalized weight stigma will be examined as a potential mediator of the relationship. Results of a cross-sectional survey showed that 74.6% of adults report they have intentionally tried to lose weight. Amongst those who have tried to lose to weight, the average number of weight cycles over the lifetime was 7.82 cycles. Simultaneous regression showed that greater weight cycling was related to greater reported depressive symptoms (β = .15, *p* < .001), controlling for age, gender, education, income, and body mass index. Internalized weight stigma was a partial mediator of this relationship. Discussion focuses on the potential implications for weight cycling and mental health.

## Introduction

As the prevalence of obesity rises in the United States and worldwide [1], increasing attention has focused on weight management. Reviews of randomized clinical trials of weight loss interventions show a consistent pattern: People initially lose weight; the weight loss plateaus around six months; slow, steady weight gain follows [2, 3]. In many cases, people gain back more weight than they lost, leaving them at a higher weight than before their initial weight loss attempt (for review see [4]). Despite, or perhaps because of, this weight regain, people attempt a new diet or exercise program, ever hopeful that “this time” they will lose the weight and keep it off [5]. Thus, people can find themselves “weight cycling” or “yo-yo dieting”–losing weight followed by gaining weight repeatedly over time.

In the current work, we focus on the potential relationship between weight cycling and mental health, specifically depressive symptomatology. Research published primarily before the year 2000 concluded that there was no relationship between weight cycling and depression [6–8]. Reviews found that once current body mass index (BMI) was taken into account, initial relationships observed between weight cycling and poorer mental health outcomes tended to disappear [9, 10]. However, these studies were conducted two decades ago, and millions of Americans continue to participate in weight loss attempts and programs every year [11]. Indeed, levels of body weight have changed dramatically in just a few decades. For example, from 1999–2016 American children ages 2–19 show increased prevalence of overweight and obesity at every age group [12]. The increasing weight of adults is also well-documented, with the prevalence of obesity increasing from 30.5% in 1999 to 42.4% in 2017 [1]. In short, more people are now living with extra body weight at an earlier age and over the lifetime.

As people are living with heavier weight across their lifespans, they are also self-reporting more frequent attempts at weight loss, with a linear upward trend in dieting from 1999 to 2016 [13]. Given the evidence of increased weight and increased weight loss attempts, we examined the relationship between weight cycling and depressive symptoms in a large sample of American adults. Two recent studies point to a connection between weight cycling and depressive symptoms. Researchers studying a representative population sample of people in Germany, found that “severe” weight cyclers (defined in this study as at least 3 cycles of losing 10 kilograms or approximately 22 pounds or more) reported higher depression levels than non-severe-weight-cyclers [14]. This study did not report results while controlling for BMI and only included participants with overweight and obese BMIs. A prospective study of mid-life women in Australia, which did control for BMI, showed higher odds of depressive symptoms for more frequent weight cyclers [15]. Thus, recent evidence suggests that weight cycling may be related to depressive symptoms. Additional research is warranted to further examine this relationship, including possible mechanisms for why these positive associations may emerge.

Research on weight stigma points to one possible pathway that may help explain the association between weight cycling and depressive symptoms: Internalized weight stigma. Internalized weight stigma, also called weight bias internalization, is the extent to which people believe that negative weight-related stereotypes (e.g., people with larger bodies have less willpower, are less competent, or are unattractive) are true of the self, and engage in self-devaluation for one’s weight status [16]. Internalized weight stigma has been consistently related to worse mental health outcomes in both treatment seeking [17] and non-treatment seeking samples [18, 19] but has not been examined in the context of weight cycling. We hypothesize that internalized weight stigma could be one pathway through which repeated weight cycling may negatively impact mental health. Specifically, repeated weight cycling may lead people to feel like they have failed to effectively control their weight and thus be more likely to internalize negative weight-related beliefs about the self.

The present study utilized a diverse, online, community sample of American adults with approximately equal numbers of men and women. The sample encompasses a broad weight and age range, and is not a treatment-seeking sample. Thus, this sample is likely more typical of the population of Americans trying to manage their weight compared to tightly controlled clinical trials of weight loss and maintenance. We expected to find a positive relationship between weight cycling and increased depressive symptoms, but, based on previous research, that this relationship may attenuate once BMI is taken into account. In addition, because previous research has found that women report more dieting than men [20] and more discrimination based on their weight [21] we assessed whether the relationship between weight cycling and depressive symptoms is moderated by gender. Finally, we examined internalized weight stigma as a mediator of the relationship between weight cycling and depressive symptoms.

## Methods

Research methods were approved by the University of Connecticut Institutional Review Board (Protocol #X15-093), with written (online) consent.

### Participants

A sample of 3087 American adults was randomly drawn from a national survey panel administered during 2015–2016 by Survey Sampling International (SSI; Shelton, CT), which includes over 2 million active research respondents recruited from more than 3400 sources. SSI employs validation processes comparing respondent demographic characteristics to multiple databases, and uses demographic quotas for sex, race, and income to approximate U.S. census data within the online population. All panelists were 18 years or older (see also [22, 23]). Participants with missing or improbable values on key variables such as height and/or weight (*n* = 312), as well as statistical outliers on these variables (*n* = 73), were excluded, resulting in a final sample of 2702 participants. The number of participants in each of the analyses below varies with missing data (people who skipped questions).

### Measures

#### Demographics

Participants reported their gender (50.7% female), age in years (*M* = 44.77, *SD* = 16.99), and racial/ethnic classification (64.1% White, not Hispanic, 15.6% Latino/Hispanic; 12.5% African American; 5.8% Asian; 2% Other). Their highest level of education was measured in categories ranging from *less than high school* to *postgraduate degree* (median education level: *college graduate*) and their current household income was measured from < $25,000 to $125,000+ in sets of $25,000 (median income was between $50,000–75,000). They also reported their current height and weight, and these numbers were used to compute their current body mass index (BMI, *M* = 26.95, *SD* = 6.02); with 5% classified as ‘underweight’ (BMI < 18.5); 37% ‘normal weight’ (BMI 18.5–24.9); 32% ‘overweight’ (BMI 25–29.9); and 25% ‘obese’ (BMI > 30).

#### Weight cycling history

Participants were first asked “Have you ever done anything to try to lose weight?” (*yes*/*no*). Those who said “yes” were presented with a weight cycling measure from Venditti and colleagues [24]. The instructions asked participants to “Please indicate the number of times you have intentionally lost the following amount of weight **in your lifetime**:” This was followed by a table with choices including 1 to 9 lbs; 10–19 lbs; 20–49 lbs; 50–79 lbs; 80–99 lbs; and 100 or more pounds. For each weight loss amount, participants indicated the number of times that they had lost that amount of weight, choosing from the categories: *Never*, *1–2 times*, *3–5 times*, *6–10 times*, *More than 10 times*. Total number of weight cycles was calculated by summing the midpoint of the number of times endorsed (0, 1.5, 4, 8, or 10) across all the weight loss magnitude categories. Previous research has also examined just the number of weight cycles of 10 pounds or more [25], and/or 20 pounds or more [26]. We calculated these as well, using the same methods as for total weight cycles.

#### Depressive symptoms

Participants completed the 10-item Center for Epidemiologic Studies–Depression scale (CES-D-10; [27, 28]). The CES-D-10 measures the frequency of common depression symptoms (such as depressed affect and somatic symptoms) during the past week (response options range from 1 = *rarely or none of the time*, to 4 = *most or all of the time*), with higher scores indicative of greater severity of depression symptoms. This measure has been well-validated with adult samples (current sample: *M* = 1.84; *SD* = .68; *α* = .89).

#### Internalized weight bias

The Weight Bias Internalization Scale–Modified (WBIS-M; [29, 30]) was completed as the measure of internalized weight stigma, which assesses endorsement of weight-related stereotypes toward oneself and self-devaluation due to weight. In line with recommendations based on the psychometric properties of the scale [31] the WBIS-M is a 10-item Likert scale with responses rated from 1 = *strongly disagree* to 7 = *strongly agree* on items such as “I hate myself for my weight” and “I don’t feel that I deserve to have a really fulfilling social life, because of my weight.” The measure has been well-validated with adult samples (current sample: *M* = 3.43; *SD* = 1.60; *α* = .94).

### Statistical analysis

We first present descriptive statistics examining the extent to which participants report ever intentionally trying to lose weight. Then, amongst those participants who report ever intentionally trying to lose weight, we examine mean levels of weight cycling reported over the lifetime as well as gender comparisons of mean levels of weight cycling. To examine the potential relationship between weight cycling and depressive symptoms, we present bivariate correlations between all of the variables followed by a simultaneous linear regression predicting depressive symptoms from weight cycling, while controlling for demographic variables, including BMI. We then examine whether the relationship between weight cycling and depressive symptoms is moderated by gender. Finally, after examining the bivariate relationships between weight cycling, internalized weight stigma, and depressive symptoms, we examine a mediation model using Hayes’ PROCESS model [32].

All analyses examining weight cycling included only those participants who reported having ever intentionally attempted weight loss (*N* = 2013), excluding those who had never intentionally tried to lose weight (*N* = 686). To facilitate comparisons of our findings to previous work on weight cycling, we present analyses using the total number of weight cycles, weight cycles of 10 or more pounds, and weight cycles of 20 or more pounds, given that previous research has used each of these definitions for weight cycling. Our results are similar regardless of which weight cycling definition is examined.

## Results

As can be seen in Table 1, almost 75% of the sample indicated that they had attempted to lose weight at least once in their lifetime. Examining these results by demographic variables shows some differences between groups. Specifically, a greater proportion of women than men indicated ever intentionally trying to lose weight; African Americans and Asian Americans were less likely than White and Latinx participants to report ever intentionally trying to lose weight. People with less education and lower income were less likely than those with higher education levels and higher income to report they had ever intentionally tried to lose weight. These group differences, however, were relatively small as indicated by both the absolute percentages and the small effect sizes (Cramer’s V) in Table 1. The majority of people in every group indicated that they had intentionally tried to lose weight at least once in their life.

**Table 1:** Self-report of whether participants had ever attempted to lose weight.

	*n*	Yes (%)	χ^2^	*p*	Cramer’s V
Sex	2691		64.55	< .001	0.16
Women	1368	81.2			
Men	1323	67.7			
Race/Ethnicity	2695		54.02	< .001	0.14
White	1730	78.2			
African American	336	64.3			
Hispanic/Latino	420	75.2			
Asian/Pacific Islander	156	59.0			
Other	53	64.2			
Education	2686		39.18	< .001	0.12
High School or less	463	63.3			
Vocational/some college	848	76.1			
College graduate and up	1375	77.7			
Current Income	2687		25.05	< .001	0.10
< 25K	408	67.2			
25K-49K	680	71.5			
50K-74	565	76.8			
75–99	461	77.4			
100–124	214	78.0			
125+	359	79.7			
**Overall**	**2699**	**74.6**	**652.4**	**< .001**	

Group differences tested using Chi-square goodness of fit statistic. Cramer’s V is a measure of effect size.

### Weight cycling

The total number of weight cycles ranged from 0 (people who said they have tried to lose weight but did not lose any) to 60, with a mean number of 7.82 weight cycles over the lifetime *(Median* = 5.5; *SD* = 8.0). The mean number of weight cycles of 10 or more pounds was 4.17 (*Median* = 1.5, *SD* = 6.18) and the mean number of weight cycles of 20 or more pounds was 2.1 (*Median* = 1.5; *SD* = 4.70).

#### Gender comparisons in weight cycling frequency

ANCOVAs examining gender differences in frequencies of weight cycles (covarying age and current BMI) found few differences between women and men. This was true for total weight cycles (*M*_women_ = 8.05, *SD*_women_ = 8.10; *M*_men_ = 7.53, *SD*_men_ = 7.83; *F*(1, 1957) = 4.12, *p* = .043, η_p_^2^ = .002), weight cycles of 10 pounds or more (*M*_women_ = 4.25, *SD*_women_ = 6.24; *M*_men_ = 4.06, *SD*_men_ = 6.13; *F*(1, 1854) = 1.41, *p* = .235, η_p_^2^ = .001), and for weight cycles of 20 pounds or more (*M*_women_ = 2.10, *SD*_women_ = 4.72; *M*_men_ = 2.10, *SD*_men_ = 4.74; *F*(1, 1757) = .11, *p* = .740, η_p_^2^ < .001). BMI was a significant covariate in each ANCOVA, with higher BMI related to more weight cycles.

### Bivariate correlations

Table 2 shows bivariate correlations between gender, age, education, income, BMI, weight cycling, and internalized weight stigma, and depressive symptoms (CES-D-10). As predicted, weight cycling was correlated with depressive symptoms, with *r*-values ranging from .12 to .15, and well as with internalized weight stigma, *r*-values ranging from .16 to .21. Replicating previous findings, gender was correlated with depressive symptoms (*r* = -.10), with women reporting more depressive symptoms than men. BMI was also correlated with depressive symptoms, (*r* = .07), with people with higher current BMIs reporting higher levels of depressive symptoms. All of the demographic variables (age, education, and income) were correlated with depressive symptoms but not with weight cycling. People who were older, wealthier, and more educated reported lower levels of depressive symptoms. All of the variables were correlated with internalized weight stigma; participants who were women, younger, less educated, lower income, higher in BMI, and who reported more frequent weight cycling all reported greater internalized weight stigma. Internalized weight stigma was strongly correlated with depressive symptomology (*r* = .62).

**Table 2:** Bivariate correlations.

	1	2	3	4	5	6	7	8	9
1: Gender	-								
2: Age	.095**	-							
3: Education	.09**	.109**	-						
4: Income	.094**	.086**	.416**	-					
5: BMI	.064**	.153**	-0.031	-.064**	-				
6: Total WC	-0.036	0.014	0.042	0.017	.167**	-			
7: ≥ 10 lb. WC	-0.019	0.028	0.018	-0.013	.163**	.914**	-		
8: ≥ 20 lb. WC	-0.002	0.02	-0.001	-0.027	.120**	.778**	.923**	-	
9. WBIS	-.128**	-.254**	-.057**	-.095**	.293**	.217**	.209**	.163**	-
10: CES-D-10	-.103**	-.296**	-.140**	-.200**	.069*	.152**	.156**	.124**	.616**

Gender is coded women (-1), men (1); BMI = Body Mass Index; Total WC = total number of weight cycles over the lifetime; ≥ 10 lb. WC = number of weight cycles with 10 pounds or more lost/regained; ≥ 20 lb. WC = number of weight cycles with 20 pounds or more lost/regained; WBIS = Weight Bias Internalization Scale-Modified; CES-D-10 = Center for Epidemiologic Studies–Depression 10 item scale.

*p < .05

** *p* < .01.

### Weight cycling and depressive symptoms

In previous research, the relationship between frequency of weight cycling and depression reduced or disappeared once BMI was taken into account [24]. Thus, we conducted a simultaneous linear regression with total lifetime weight cycles predicting depressive symptoms with gender, age, education, income, and BMI all entered as covariates. As depicted in the first column of Table 3, weight cycling remained a significant predictor of depressive symptoms with all of the covariates in the model (full model *F*(6, 1731) = 52.16, *p* < .001). Similar patterns were found when examining weight cycles of 10 pounds or more (column 2 in Table 3), and weight cycles 20 pounds or more (column 3 in Table 3).

**Table 3:** Predicting depressive symptoms (CES-D-10) from weight cycling.

	Total weight cycles (N = 1737)		Weight cycles of 10 lbs or more (N = 1647)		Weight cycles of 20 lbs of more (N = 1563)	
	β	*p*	β	*p*	β	*p*
Gender	-0.03	0.181	-.03	.223	-.04	.130
Age	-0.27	< .001	-.27	< .001	-.27	< .001
Education	-0.06	0.016	-.05	.037	-.04	.094
Income	-0.17	< .001	-.16	< .001	-.17	< .001
BMI	0.05	0.049	.05	.060	.06	.019
**Weight Cycling**	0.15	< .001	.15	< .001	.11	< .001
**Total Adj. R**^**2**^	**0.15**	**< .001**	**0.145**	**< .001**	**.138**	**< .001**
Gender X Weight Cycling Interaction	-.05	.121	-.04	.164	-.02	.423

#### Interaction between gender and weight cycling on depressive symptoms

In order to examine whether the relationship between weight cycling and depressive symptoms was moderated by gender, we added the interaction term to the linear regressions reported above. As can been seen in the final row of Table 3, the interaction was not significant, suggesting that the effect of weight cycling on depressive symptoms is similar for men and women in this sample.

#### Mediation of weight cycling and depressive symptoms by internalized weight stigma

**Fig 1:**
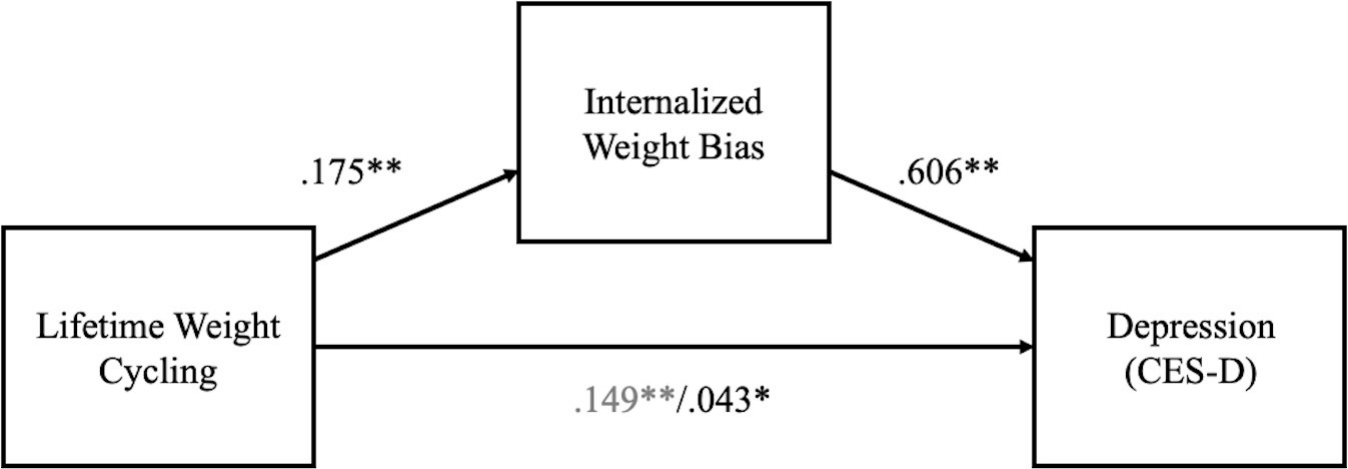
The relationship between weight cycling and depressive symptoms mediated by internalized weight stigma. Path weights are standardized regression weights. Total effect is printed in grey text. Mediation analysis included gender, age, education, income, and BMI as covariates.

Using Hayes [32] PROCESS macro (version: 3.4.1), we examined whether the relationship between weight cycling and depressive symptoms was mediated by internalized weight stigma. Gender, age, education, income, and BMI were included as covariates in the analysis. As can be seen in Fig 1, the direct effect of weight cycling on depressive symptoms was reduced (but not eliminated) by the inclusion of internalized weight bias in the model. The indirect effect of weight cycling on depressive symptoms through internalized weight bias was significant (standardized *b* = .106; *SE* = .01; 95% CI [.079, .133]), such that greater number of weight cycles was related to increased internalized weight stigma, which in turn predicted greater reported depressive symptoms. The full model accounted for 45% of the variance in depressive symptoms.

## Discussion

Our study examined the relationship between weight cycling over the lifetime and depressive symptoms and whether internalized weight stigma is a potential pathway linking weight cycling and adverse mental health. The mediating role of internalized weight stigma in the relationship between weight cycling and depressive symptoms has been neglected in the literature to date, despite an emergence of evidence documenting internalized weight stigma and its negative implications for weight-related health and psychological wellbeing. Our findings begin to address this research gap, and offer important insights suggesting that frequent weight cycling may be associated with greater depressive symptoms for American adults, and that internalized weight stigma is a partial mediator of this relationship.

In line with recent estimates suggesting that dieting is common among adults in the U.S [20], approximately 75% of the present sample reported that they had intentionally tried to lose weight at least once in their lifetime, with an average of 7.8 weight cycles of losing and gaining weight. Weight cycling was associated with greater depressive symptoms, and this relationship was not negated by the inclusion of current BMI in the analyses. Although more women than men reported ever intentionally trying to lose weight, amongst those who had attempted weight loss there were no gender differences in the frequency of weight cycling and gender did not moderate the relationship between weight cycling and depressive symptoms.

Given the relationship found between weight cycling and depressive symptoms, we explored internalized weight stigma as a potential mediator of this relationship. Previous work has found internalized weight stigma to be strongly correlated with depressive symptoms [18, 19] and that finding was replicated in the current sample. A recent longitudinal study of weight-loss maintainers [33] found that internalized stigma decreased over time with weight loss, showing that changes in weight can predict changes in internalized weight stigma. Current analyses supported internalized weight stigma as a mediator of the relationship between weight cycling and depressive symptoms. Thus, one potential explanation for how repeated weight cycling impacts depression is through its positive relationship with internalized weight stigma–the more frequently people lose and then regain the weight, the more they internalize negative beliefs about the self, the more frequent their depressive symptoms. It is important to note that this relationship is likely part of a larger cycle not captured here: both dieting and stigma are stressful, theoretically increasing the likelihood of using food to cope, and therefore impacting weight regain [34]. If captured over time, the relationship between weight cycling and internalized weight stigma is likely to be bidirectional. Moreover, analyses showed that the mediation of the weight cycling to depressive symptoms path by internalized weight stigma was partial, meaning that there are additional variables, not measured here, that also help account for the relationship.

The current research used a large, diverse sample to inform understanding of the relationship between weight cycling and depressive symptoms in American adults. This sample is likely more typical of Americans who attempt to lose weight than participants in tightly-controlled clinical trials for weight loss, increasing the generalizability of our findings. In addition, this study provides novel insights about potential links between weight cycling and internalized weight stigma, a relationship that has received little attention in the literature. Our findings suggest that this relationship warrants additional research to better understand the role of internalized weight stigma and its relevance to weight cycling.

There are several limitations of this study. First, given the cross-sectional nature of the survey, directionality and causality between weight cycling and depressive symptoms cannot be assumed. It is possible that changes in depressive symptoms drive weight cycling. Depression has been related to both weight gain and weight loss (DSM-V; [35]). However, multi-year tracking in a weight loss intervention study found that increased depressive symptoms did not drive weight re-gain [36]. Moreover, the accuracy of mediation effects in cross sectional data have been questioned [37]. Collecting longitudinal data would be a stronger and more accurate test of directionality and mediation. Second, the study relies on retrospective reporting of weight loss cycles. Although this is common in the literature, it is difficult to determine how accurately people recall each of their weight loss attempts and the amount of weight lost, especially when considered over the lifetime. Third, although we examined gender as moderator in our analyses, we do not have the statistical power to examine the intersection of gender and ethnicity in our results. Given recent evidence of differences in internalized weight stigma across ethnic and gender identities [22], it will be important to examine the links between internalization, psychological well-being, and weight cycling in larger samples with more racial and ethnic diversity. Fourth, the obesity rate in the current sample (25%) was lower than the national rate (39%) at the time data collection [38]. Thus, it is possible that the amount of weight cycling reported here is lower than the actual rate, reducing the generalizability of the study. Whereas our study findings need to be replicated in larger samples of people who have obesity, our results nevertheless suggest that links between internalized stigma, weight cycling, and depressive symptoms may be relevant for people across diverse weight categories and persist after controlling for BMI. Finally, this is self-report data with all the caveats therein.

Body weight has been increasing in both children and adults over the last several decades [1, 12]. Although these societal weight changes have garnered considerable attention to obesity related health issues, comparatively little attention has been given to the psychological effects of repeatedly attempting to reduce weight. Whereas a recent review concluded there was no evidence that intentional weight cycling was related to early mortality [39], the current findings suggest that frequent weight cycling may be linked to greater depressive symptoms for American adults. These findings indicate the importance of taking mental health into account when considering the efficacy of weight loss, and reiterate the need for health care providers and practitioners to balance the potential physical health benefits of intentional weight loss with the potential psychological consequences associated with internalized weight stigma and repeated weight cycling.

## References

[1] Hales CM, Carroll MD, Fryar CD, Ogden CL. Prevalence of obesity among adults and youth: United States, 2015–2016. 2017. 29155689

[2] Franz MJ, VanWormer JJ, Crain AL, Boucher JL, Histon T, Caplan W, et al Weight-loss outcomes: a systematic review and meta-analysis of weight-loss clinical trials with a minimum 1-year follow-up. J Am Diet Assoc 2007;107(10):1755–1767. 17904936 10.1016/j.jada.2007.07.017

[3] MacLean PS, Wing RR, Davidson T, Epstein L, Goodpaster B, Hall KD, et al NIH working group report: innovative research to improve maintenance of weight loss. Obesity 2015;23(1):7–15. 25469998 10.1002/oby.20967 PMC5841916

[4] Mann T, Tomiyama AJ, Westling E, Lew A, Samuels B, Chatman J. Medicare's search for effective obesity treatments: diets are not the answer. Am Psychol 2007;62(3):220. 17469900 10.1037/0003-066X.62.3.220

[5] Polivy J, Herman CP. If at first you don't succeed: False hopes of self-change. Am Psychol 2002;57(9):677. 12237978

[6] Bartlett SJ, Wadden TA, Vogt RA. Psychosocial consequences of weight cycling. J Consult Clin Psychol 1996;64(3):587. 8698953 10.1037//0022-006x.64.3.587

[7] Kuehnel RH, Wadden TA. Binge eating disorder, weight cycling, and psychopathology. Int J Eat Disord 1994;15(4):321–329. 8032347 10.1002/eat.2260150403

[8] Simkin‐Silverman LR, Wing RR, Plantinga P, Matthews KA, Kuller LH. Lifetime weight cycling and psychological health in normal‐weight and overweight women. Int J Eat Disord 1998;24(2):175–183. 9697016 10.1002/(sici)1098-108x(199809)24:2<175::aid-eat7>3.0.co;2-b

[9] Friedman MA, Schwartz MB, Brownell KD. Differential relation of psychological functioning with the history and experience of weight cycling. J Consult Clin Psychol 1998;66(4):646. 9735581 10.1037//0022-006x.66.4.646

[10] National Task Force on the Prevention and Treatment of Obesity. Dieting and the development of eating disorders in overweight and obese adults. Arch Intern Med 2000 9 25;160(17):2581–2589. 10999971 10.1001/archinte.160.17.2581

[11] Gudzune KA, Doshi RS, Mehta AK, Chaudhry ZW, Jacobs DK, Vakil RM, et al Efficacy of commercial weight-loss programs: an updated systematic review. Ann Intern Med 2015;162(7):501–512. 25844997 10.7326/M14-2238 PMC4446719

[12] Skinner AC, Ravanbakht SN, Skelton JA, Perrin EM, Armstrong SC. Prevalence of Obesity and Severe Obesity in US Children, 1999–2016. Pediatrics 2018 3;141(3): 10.1542/peds.2017-3459 PMC6109602 29483202

[13] Han L, You D, Zeng F, Feng X, Astell-Burt T, Duan S, et al Trends in Self-perceived Weight Status, Weight Loss Attempts, and Weight Loss Strategies Among Adults in the United States, 1999–2006. JAMA network open 2019;2(11):e1915219–e1915219. 31722029 10.1001/jamanetworkopen.2019.15219 PMC6902793

[14] de Zwaan M, Engeli S, Müller A. Temperamental factors in severe weight cycling. A cross-sectional study. Appetite 2015;91:336–342. 25931432 10.1016/j.appet.2015.04.064

[15] Madigan CD, Pavey T, Daley AJ, Jolly K, Brown WJ. Is weight cycling associated with adverse health outcomes? A cohort study. Prev Med 2018;108:47–52. 29277416 10.1016/j.ypmed.2017.12.010

[16] Durso LE, Latner JD. Understanding self‐directed stigma: development of the weight bias internalization scale. Obesity 2008;16(S2):S80–S86. 18978768 10.1038/oby.2008.448

[17] Carels RA, Wott CB, Young KM, Gumble A, Koball A, Oehlhof MW. Implicit, explicit, and internalized weight bias and psychosocial maladjustment among treatment-seeking adults. Eating Behaviors 2010 8 2010;11(3):180–185. 10.1016/j.eatbeh.2010.03.002 20434066

[18] Lee MS, Gonzalez BD, Small BJ, Thompson JK. Internalized weight bias and psychological wellbeing: An exploratory investigation of a preliminary model. PLoS One 2019 5 9;14(5):e0216324. 31071115 10.1371/journal.pone.0216324 PMC6508719

[19] Pearl RL, Puhl RM. Weight bias internalization and health: a systematic review. Obesity reviews 2018;19(8):1141–1163. 29788533 10.1111/obr.12701 PMC6103811

[20] Saad L. To lose weight, Americans rely more on dieting than exercise.Gallup Poll Social Series: Health and Healthcare; 2011 2014.

[21] Spahlholz J, Baer N, König H, Riedel‐Heller S, Luck‐Sikorski C. Obesity and discrimination–a systematic review and meta‐analysis of observational studies. Obesity reviews 2016;17(1):43–55. 26596238 10.1111/obr.12343

[22] Himmelstein MS, Puhl RM, Quinn DM. Intersectionality: an understudied framework for addressing weight stigma. Am J Prev Med 2017;53(4):421–431. 28579331 10.1016/j.amepre.2017.04.003

[23] Puhl RM, Quinn DM, Weisz BM, Suh YJ. The role of stigma in weight loss maintenance among US adults. Annals of Behavioral Medicine 2017;51(5):754–763. 28251579 10.1007/s12160-017-9898-9

[24] Venditti EM, Wing RR, Jakicic JM, Butler BA, Marcus MD. Weight cycling, psychological health, and binge eating in obese women. J Consult Clin Psychol 1996;64(2):400–405. 8871424 10.1037//0022-006x.64.2.400

[25] Stevens VL, Jacobs EJ, Sun J, Patel AV, McCullough ML, Teras LR, et al Weight cycling and mortality in a large prospective US study. Am J Epidemiol 2012;175(8):785–792. 22287640 10.1093/aje/kwr378

[26] Osborn RL, Forys KL, Psota TL, Sbrocco T. Yo-yo dieting in African American women: weight cycling and health. Ethn Dis 2011 Summer;21(3):274–280. 21942158 PMC3963267

[27] Radloff LS. The CES-D Scale: A self-report depression scale for research in the general population. Applied Psychological Measurement 1977;1(3):385–401.

[28] Irwin M, Artin KH, Oxman MN. Screening for depression in the older adult: criterion validity of the 10-item Center for Epidemiological Studies Depression Scale (CES-D). Arch Intern Med 1999;159(15):1701–1704. 10448771 10.1001/archinte.159.15.1701

[29] Durso LE, Latner JD, Ciao AC. Weight bias internalization in treatment-seeking overweight adults: Psychometric validation and associations with self-esteem, body image, and mood symptoms. Eating Behav 2016;21:104–108. 10.1016/j.eatbeh.2016.01.011 26826975

[30] Pearl RL, Puhl RM. Measuring internalized weight attitudes across body weight categories: validation of the modified weight bias internalization scale. Body image 2014;11(1):89–92. 24100004 10.1016/j.bodyim.2013.09.005

[31] Lee M, Dedrick RF.Weight bias internalization scale: psychometric properties using alternative weight status classification approaches. Body Image 2016;17: 25–29. 26907913 10.1016/j.bodyim.2016.01.008

[32] Hayes AF. Introduction to Mediation, Moderation, and Conditional Process Analysis: A Regression-Based Approach. 2nd ed. New York, NY: Guilford Press; 2018.

[33] Olson KL, Lillis J, Graham Thomas J, Wing RR. Prospective evaluation of internalized weight bias and weight change among successful weight‐loss maintainers. Obesity 2018;26(12):1888–1892. 30421843 10.1002/oby.22283 PMC6249105

[34] Tomiyama AJ. Weight stigma is stressful. A review of evidence for the Cyclic Obesity/Weight-Based Stigma model. Appetite 2014;82:8–15. 24997407 10.1016/j.appet.2014.06.108

[35] American Psychiatric Association. Depressive disorders. Diagnostic and statistical manual of mental disorders. 5th ed.; 2013.

[36] Price DW, Ma Y, Rubin RR, Perreault L, Bray GA, Marrero D, et al Depression as a predictor of weight regain among successful weight losers in the diabetes prevention program. Diabetes Care 2013 2;36(2):216–221. 23002085 10.2337/dc12-0293 PMC3554307

[37] Maxwell SE, Cole DA. Bias in cross-sectional analyses of longitudinal mediation. Psychol Methods 2007;12(1):23. 17402810 10.1037/1082-989X.12.1.23

[38] Hales CM, Carroll MD, Fryar CD, Ogden CL. Prevalence of obesity and severe obesity among adults: United States, 2017–2018 NCHS Data Brief, no 360. Hyattsville, MD: National Center for Health Statistics 2020 32487284

[39] Mehta T, Smith DL Jr, Muhammad J, Casazza K. Impact of weight cycling on risk of morbidity and mortality. Obesity reviews 2014;15(11):870–881. 25263568 10.1111/obr.12222 PMC4205264

